# Terahertz spectroscopy of 2,4,6-trinitrotoluene molecular solids from first principles

**DOI:** 10.3762/bjoc.14.26

**Published:** 2018-02-09

**Authors:** Ido Azuri, Anna Hirsch, Anthony M Reilly, Alexandre Tkatchenko, Shai Kendler, Oded Hod, Leeor Kronik

**Affiliations:** 1 Department of Materials and Interfaces, Weizmann Institute of Science, Rehovoth 76100, Israel; 2 School of Chemical Sciences, Dublin City University, Glasnevin, Dublin 9, Ireland; 3 Physics and Materials Research Unit, University of Luxembourg, L-1511 Luxembourg; 4 Israel Institute for Biological Research, Ness Ziona 74100, Israel; 5 Department of Physical Chemistry, School of Chemistry, The Raymond and Beverly Sackler Faculty of Exact Sciences and The Sackler Center for Computational Molecular and Materials Science, Tel Aviv University, Tel Aviv 6997801, Israel

**Keywords:** density functional theory, THz spectroscopy

## Abstract

We present a computational analysis of the terahertz spectra of the monoclinic and the orthorhombic polymorphs of 2,4,6-trinitrotoluene. Very good agreement with experimental data is found when using density functional theory that includes Tkatchenko–Scheffler pair-wise dispersion interactions. Furthermore, we show that for these polymorphs the theoretical results are only weakly affected by many-body dispersion contributions. The absence of dispersion interactions, however, causes sizable shifts in vibrational frequencies and directly affects the spatial character of the vibrational modes. Mode assignment allows for a distinction between the contributions of the monoclinic and orthorhombic polymorphs and shows that modes in the range from 0 to ca. 3.3 THz comprise both inter- and intramolecular vibrations, with the former dominating below ca. 1.5 THz. We also find that intramolecular contributions primarily involve the nitro and methyl groups. Finally, we present a prediction for the terahertz spectrum of 1,3,5-trinitrobenzene, showing that a modest chemical change leads to a markedly different terahertz spectrum.

## Introduction

The transparency of many non-conductive materials in the terahertz range (0.1 to 10 THz) of the electromagnetic spectrum has led to the development of several potential applications of terahertz radiation [1]. Terahertz spectroscopy of molecular solids, in particular, has gained significant attention as it offers the possibility of distinguishing between different solid forms based on the signature of intermolecular vibrations [1–8]. For example, terahertz spectroscopy has been used to distinguish between different polymorphs of molecular solids used for pharmaceutical purposes [4–5,9–11], to detect contamination in food [12], in genetic [13] and skin-cancer [14] diagnosis, and in other applications [1–2,4–7].

One intriguing application of terahertz spectroscopy is the detection of energetic materials, which is of obvious importance for defense purposes [15]. Indeed, terahertz spectra were measured and analyzed for a variety of typical energetic materials, such as octahydro-1,3,5,7-tetranitro-1,3,5,7-tetraazacyclooctane (HMX), 2,4-dinitrotoluene (DNT), cyclotrimethylenetrinitramine (RDX), and pentaerythritol tetranitrate (PETN) [8,16–18]. Specifically for 2,4,6-trinitrotoluene (TNT), a very well-known energetic material, terahertz spectra were measured by several groups [16–19]. In particular, Melinger et al. [16] have obtained a high-resolution, low-temperature (12 K) spectrum at frequencies up to approx. 3.5 THz. The analysis of these spectra, especially with respect to the assignment of vibrational modes, is complicated by the fact that TNT samples typically contain at least two co-existing polymorphs, a monoclinic one (majority) and an orthorhombic one (minority). Furthermore, the relative contribution of inter- and intramolecular vibrational components remains under debate.

The above difficulties in analysis can be overcome by employing first principles calculations, in which each pure polymorph can be studied individually [20–22]. A leading first principles approach that can yield reliable simulated spectra for complex materials is density functional theory (DFT) [23]. A significant complication, however, is that conventionally used exchange–correlation energy functionals in DFT do not describe the intermolecular dispersion interactions well. Therefore, early calculations employing them were not always able to achieve satisfactory agreement with experiment, as cautioned in [24]. Recent years have seen major improvements in DFT augmented by pair-wise dispersion interaction terms, to the point where they can be used regularly to predict properties of molecular solids [21,25–26]. Indeed, simulated terahertz spectra based on dispersion-inclusive DFT were recently reported for several molecular crytals [20,27].

Here, we employ dispersion-inclusive DFT calculations based on the Tkatchenko–Scheffler (TS) approach [28] to study the terahertz spectra of TNT. These calculations are used to assign modes in both TNT polymorphs studied. They reveal modes contributed by the different polymorphs and distinguish modes dominated by intermolecular motion (at low frequencies) from modes dominated by a combination of inter- and intramolecular movement (at higher frequencies). These results are further validated by comparing them to uncorrected DFT calculations on the one hand and to more sophisticated many-body dispersion DFT calculations on the other hand. The same methodology is then used to predict the terahertz spectra of the related 1,3,5-trinitrobenzene (TNB) molecular solid, demonstrating that the elimination of the methyl group changes significant fingerprints in the terahertz spectrum.

## Computational Approach

All calculations were performed based on the generalized-gradient approximation exchange–correlation functional of Perdew, Burke and Ernzerhof (PBE) [29], with or without Tkatchenko–Scheffler–van der Waals (TS-vdW) interactions [28]. In this approach, the vdW energy is added as a pair-wise interaction and has only one semi-empirical parameter. This parameter determines the onset of the pair-wise interaction and is fitted, once and for all per a given functional, against the S22 data set of weakly bounded complexes [30]. For going beyond pair-wise interactions, we apply the many-body dispersion (MBD) method [31–32]. Within this approach, one first evaluates the TS-vdW dispersion parameters. Then, the atomic response functions are mapped onto a set of quantum harmonic oscillators that are coupled through dipole–dipole interactions to obtain self-consistent screened polarizabilities. The latter are used to calculate the correlation energy of the interacting oscillator model system, within the random-phase approximation.

Most calculations presented here were performed using VASP, a projector-augmented planewave code [33], using an energy planewave cutoff of 950 eV. Comparison to MBD calculations was performed within the CASTEP code [34], with an energy planewave cutoff of 800 eV. For both above-mentioned polymorphs of TNT, the Brillouin zone of the crystallographic unit cell was sampled using a Monkhorst–Pack *k*-grid [35] of 2 × 4 × 1. We have additionally computed the orthorhombic polymorph of TNB, using a Monkhorst–Pack *k*-point grid of 2 × 1 × 2 along the three reciprocal lattice vectors. The self-consistent cycle was converged to better than 10^−7^ eV for the total energy, to allow for numerically stable derivatives. Complete relaxation of all forces and stress components was performed prior to the calculation of vibrational frequencies. All forces in the optimized structures were smaller than 5 × 10^−3^ eV/Å, and all stress components were smaller than 0.02 GPa in the VASP and CASTEP codes.

Vibrational frequencies, *f_n_*, for each structure were calculated from *f_n_* = (1/2π)ε*_n_^1/2^*, where ε*_n_* are the eigenvalues of the mass-weighted Hessian matrix, *W*. The matrix elements of *W* are given by:

[1]
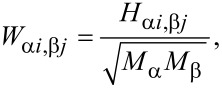


where *M*_α_ and *M*_β_ are the masses of atoms α and β, and *H*_α_*_i,_*_β_*_j_* are matrix elements of the Hessian matrix *H*, given by:

[2]
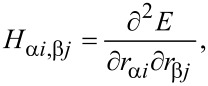


where *i*, *j* denote the Cartesian directions *x*,*y*, or *z*.

The Hessian matrix elements were determined numerically from the forces acting on the atoms for a given displacement (as computed via analytical derivatives), using the form:

[3]
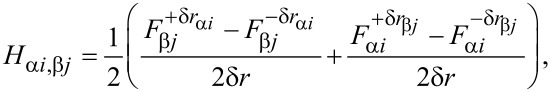


where *F* is the force acting on atom β/α in direction *j*/*i* (see subscript of *F*), as a result of atom α/β being displaced by ±δ*r* in the direction *i*/*j* (see superscript of *F*). The displacement amplitude for constructing the Hessian was chosen as 0.01 Å for TNT and 0.015 Å for TNB. These displacement amplitudes were chosen to be large enough to minimize numerical noise but small enough to minimize anharmonic contributions. We found the calculated frequencies to be numerically stable to within 0.05 THz at most and typically less than that.

The normalized eigenvectors, 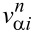, of *W* are factorized by 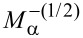 to yield the normal mode displacement eigenvectors:

[4]
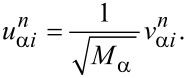


Finally, the absorption intensity *I_n_* of each mode is given by [36–41]:

[5]
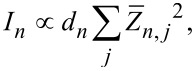


where

[6]
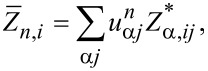


*d_n_* is the degeneracy of the mode, and


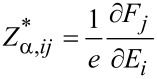


are the Born effective charge tensor elements of each atom, with *F* the force, *E* an external electric field, and *e* the electron charge.

To facilitate comparison with experiment, Lorentzian functions of the type


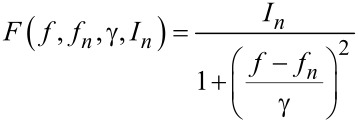


were used to broaden peaks at frequencies *f_n_* and intensities *I_n_*. We used a broadening parameter of γ = 0.0075 THz, which is similar to the measured experimental widths. The computed spectra have been scaled with respect to the highest-intensity peak observed in experiment, for the range of frequencies studied here.

## Results and Discussion

### Structural analysis

**Figure 1:**
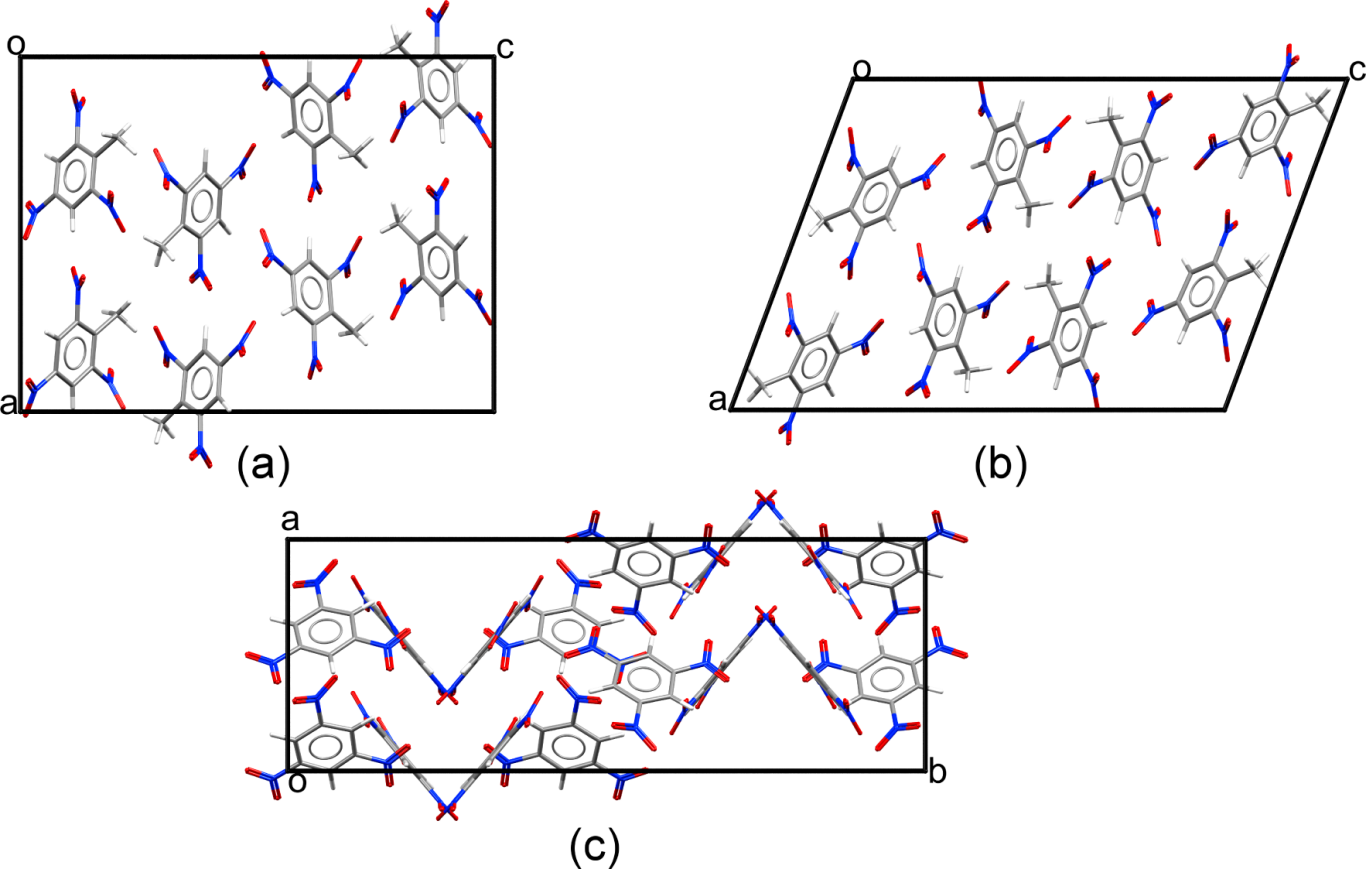
Structures of: (a) orthorhombic TNT, (b) monoclinic TNT, (c) orthorhombic TNB. For each structure, the direction of two of the three lattice vectors (*a*, *b*, *c*) is shown in the figure and the third one points inwards at the origin (*o*).

Before discussing vibrational properties, we first ascertain that our computational approach is sufficiently accurate for obtaining reliable structural predictions. Crystallographic coordinates for orthorhombic and monoclinic 2,4,6-TNT, which crystallize in the space groups *Pca*2_1_ and *P*2_1_/*a*, respectively, were obtained from [42–43]. These coordinates correspond to measurements at room temperature and 100 K, respectively, and were used as the starting point for computational structural relaxation. For 1,3,5-TNB, crystallographic data of a solid with the space group symmetry of *Pbca*, measured at room temperature, were taken from [44] and subsequently relaxed. The structures are shown in Figure 1, with a comparison between the measured and computed lattice parameters given in Table 1.

**Table 1:** Measured and computed lattice parameters for orthorhombic TNT, monoclinic TNT, and orthorhombic TNB. *a*, *b*, and *c* are lattice parameters (in Å) and β is the angle between *a* and *c*, in degrees, for the monoclinic polymorph. Numbers in parentheses indicate the relative error with respect to experiment.

		*a*	*b*	*c*	β

TNT orthorhombic	experimental [42]	14.99	6.08	20.02	
	PBE+TS-vdW	15.11 (0.8%)	6.06 (−0.33%)	19.94 (−0.4%)	
	PBE	15.65 (4.4%)	6.32 (3.95%)	21.87 (9.24%)	

TNT monoclinic	experimental [43]	14.91	6.03	20.88	110.37
	PBE+TS-vdW	15.13 (1.48%)	6.07 (0.66%)	21.17 (1.39%)	110.26 (−0.1%)
	PBE	15.66 (5.03%)	6.33 (4.98%)	23.27 (11.45%)	110.11 (−0.24%)

TNB orthorhombic	experimental [44]	9.78	26.94	12.82	
	PBE+TS-vdW	9.61 (−1.74%)	27.14 (0.74%)	12.82 (0.0%)	
	PBE	10.71 (9.51%)	27.73 (2.93%)	13.38 (4.37%)	

Clearly, excellent agreement with experiment is obtained when the PBE+TS-vdW method is used, with residual differences between theory and experiment of the order of 1%. Notably, agreement is much less satisfactory if such interactions are not included, underscoring their importance. In the absence of vdW interactions, lattice parameters are in general too large (owing to the lack of van der Waals attraction) and errors with respect to experiment are of the order of 5–10%. These observations are fully consistent with previous studies that have compared PBE and PBE+TS-vdW predictions for geometries [25,45].

### Terahertz spectra

**Figure 2:**
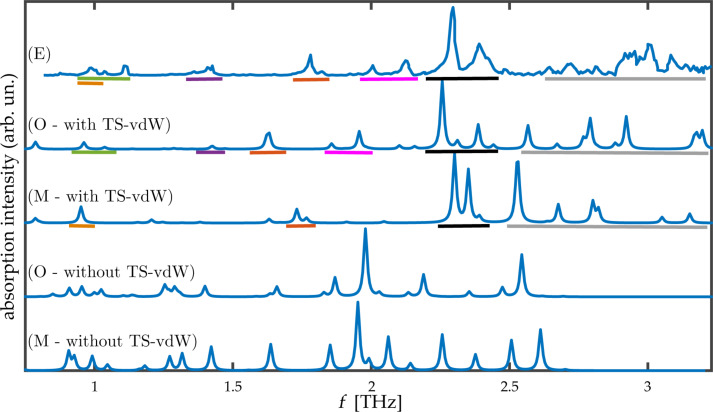
Absorption intensity (in arbitrary units) as a function of the frequency for the two TNT polymorphs studied in this work. (E) Experimental spectrum for the mixed-polymorph TNT crystal, taken from [16], as captured by a data analysis program. All other spectra are computed for: (O) the orthorhombic structure and (M) the monoclinic structure, calculated with and without TS-vdW interactions. Colored horizontal lines appearing below various experimental and computed peaks denote peak assignment.

The excellent agreement between the experimental and computed lattice parameters serves as the foundation for computing terahertz spectra. The latter, however, require not only the calculation of reliable equilibrium structures but also accurate potential energy surface curvatures, placing a more severe challenge for the TS-vdW scheme used here. The computed spectra for the two TNT polymorphs, in the range from 0 to 3.3 THz, is given in Figure 2. Several interesting observations can be drawn from the figure. First, it is readily observed that inclusion of TS-vdW interactions improves agreement with experiment dramatically, also for terahertz spectroscopy. Some discrepancies remain, e.g., the theoretical group of peaks denoted in gray, starting at ca. 2.5 THz, is slightly shifted to lower frequencies compared to experiment. Nevertheless, as shown in the lower two panels of the figure, without TS-vdW interactions various vibrational modes are strongly shifted to much lower frequencies (consistent with the missing treatment of van der Waals interactions) and, furthermore, the overall spectral shape is different.

Second, the calculation indicates that the experimental spectrum contains contributions from both the monoclinic and the orthorhombic polymorphs. Perhaps the clearest examples are the peaks at ca. 1.4 THz and ca. 2.0 THz (underlined by purple and pink, respectively, in the figure), which arise from the orthorhombic polymorph. Other important peaks, for example a major feature at ca. 2.3 THz (underlined by black in the figure), arise from both polymorphs.

**Figure 3:**
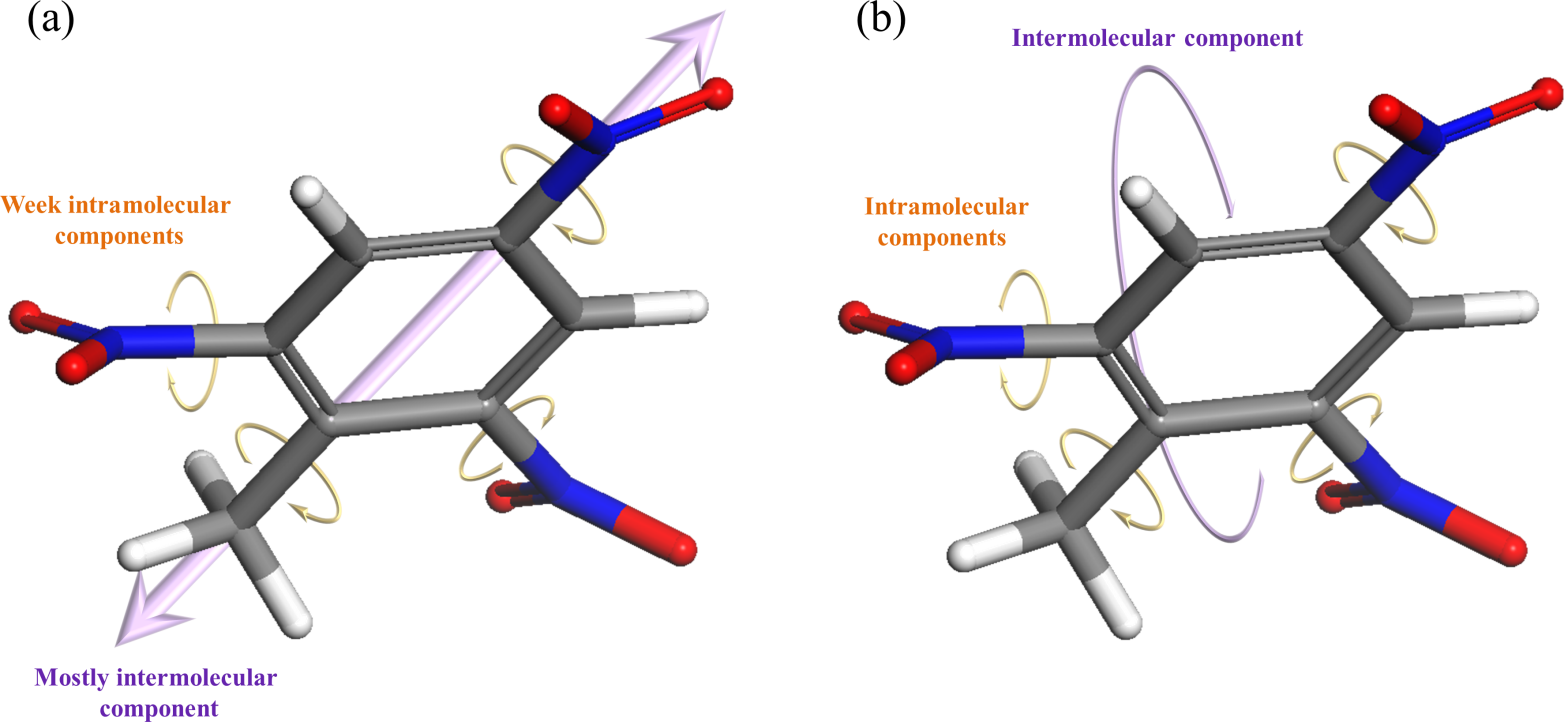
Schematic representation of typical TNT vibrational modes, demonstrated using modes of the orthorhombic polymorph at frequencies of (a) ca. 1.0 THz, (b) ca. 2.3 THz. Purple and orange arrows denote the direction of primary inter- and intramolecular displacement components.

Next, we analyze the nature of the vibrational modes, illustrated qualitatively for selected modes in Figure 3. Our analysis shows that modes below ca. 1.5 THz are mostly dominated by intermolecular vibration, whereas modes above ca. 2.0 THz possess both inter- and intramolecular vibrational components. Specifically, the dominant intramolecular motion involves torsion of nitro and methyl groups, marked by orange arrows in the figure. This is in agreement with the assignment assumed in [16], as well as with force-field calculations reported in [46]. Here, however, this assignment is obtained from first principles.

**Figure 4:**
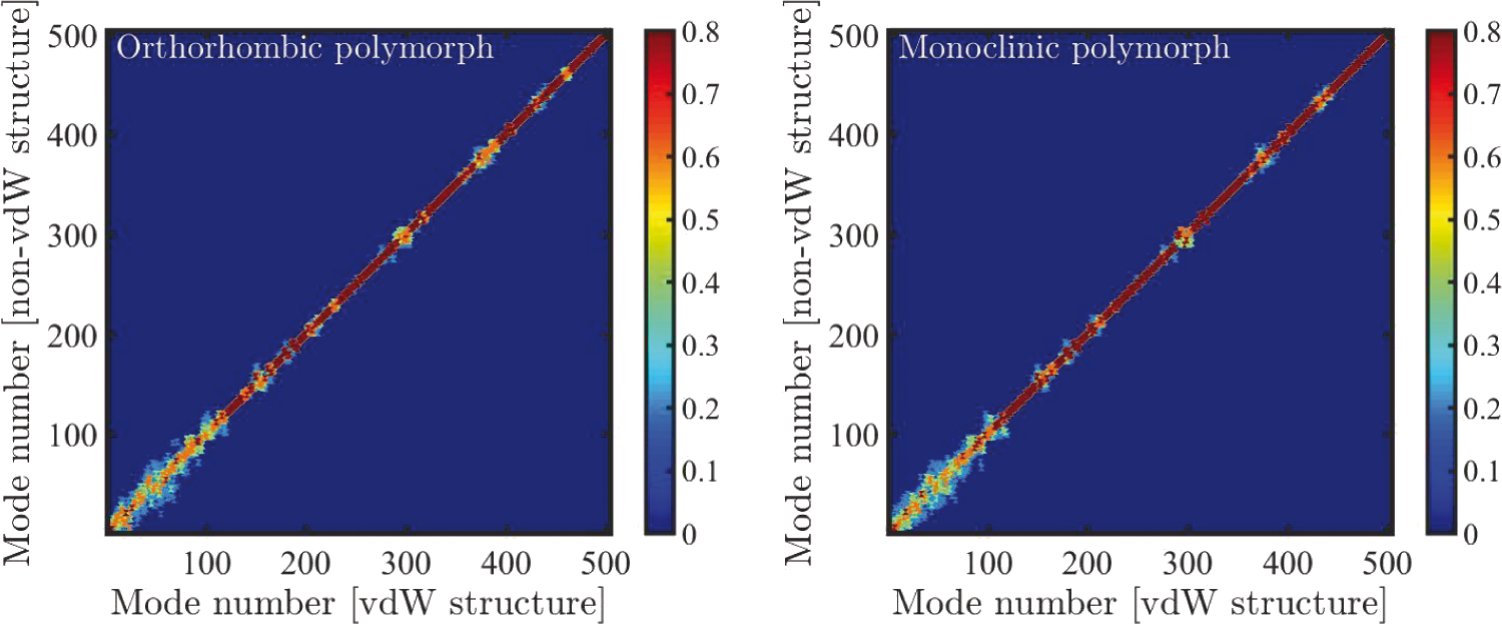
Absolute value of the scalar products between eigenvectors of the mass-weighted Hessian matrix, as obtained with and without TS-vdW pair-wise interactions for both the orthorhombic and monoclinic polymorphs of 2,4,6-trinitrotoluene. Mode no. 125, above which the eigenvectors are very similar with and without pair-wise interactions, is at ca. 8.1 THz for the dispersion-inclusive computation.

The effect of the pair-wise dispersion interactions on the vibrational modes themselves (i.e., beyond just a shift in their frequencies) can be assessed by considering the (absolute value of the) scalar product of eigenvectors of the mass-weighted Hessian matrix, with and without TS-vdW interactions. For perfectly identical modes, the multiplication should be equal to one, with the (absolute value of the) product decreasing owing to differences, down to zero for perfectly orthogonal modes. Therefore, the matrix constructed from all scalar products between modes obtained with and without TS-vdW interactions should be the identity matrix if the modes are identical. The computed matrix, arranged by mode number (in order of increasing mode frequency) for both the orthorhombic and monoclinic polymorphs, is given in Figure 4. Clearly, in the high-frequency regime (above mode 125, ca. 8 THz) the matrix elements are close to those of the identity matrix, indicating that dispersion interactions have very little direct effect on the vibrational mode. This is because these modes are mostly dominated by intramolecular motions. However, in the lower-terahertz regime (below mode 125, ca. 8 THz), which is of relevance here, significant deviations from the identity matrix arise. As discussed above, in this regime there is a non-negligible (and sometimes dominant) component of intermolecular movement. We therefore conclude that dispersion interactions are important not only for determining the vibrational frequencies but also for understanding the spatial character of the vibrational modes.

**Figure 5:**
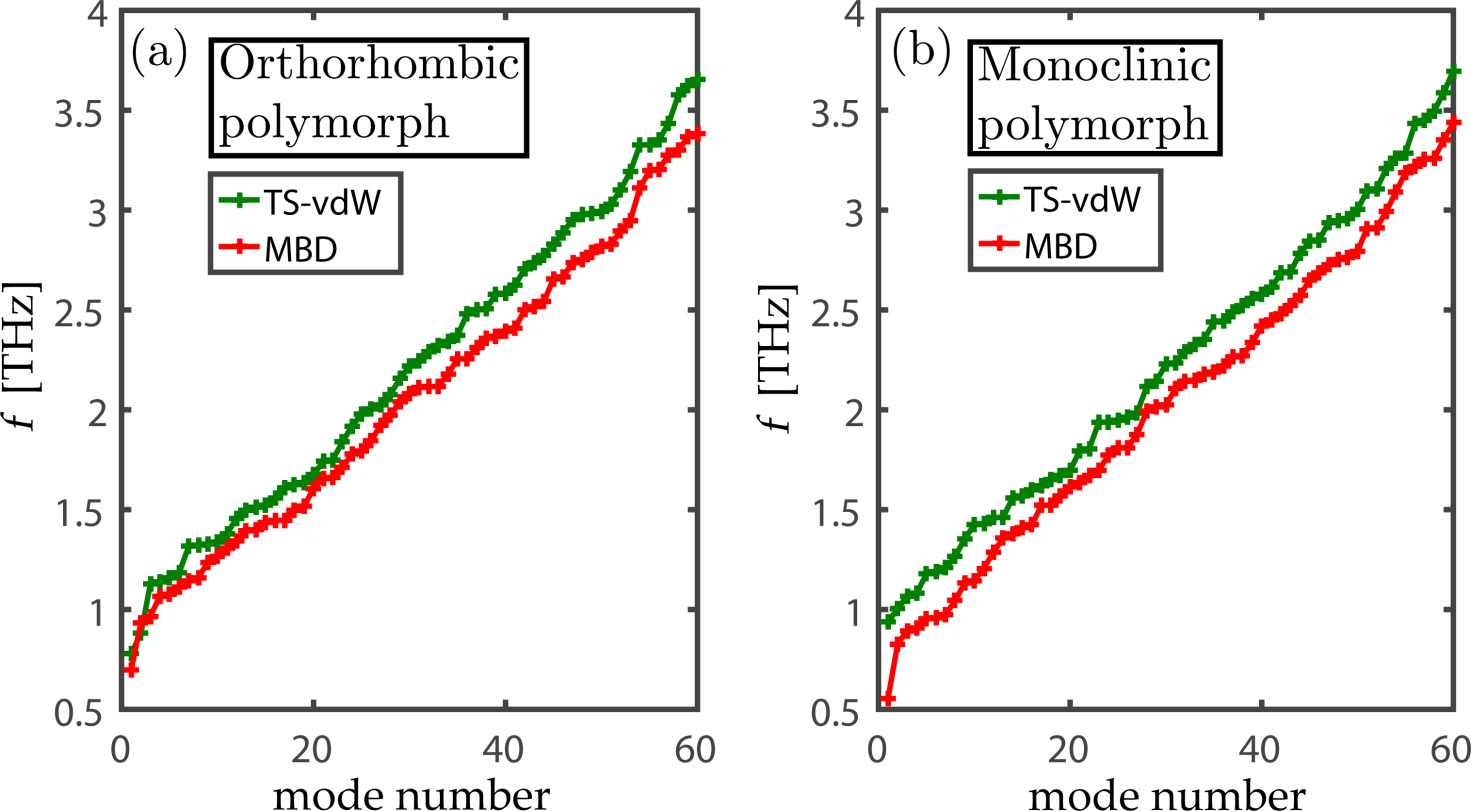
THz vibrational frequencies, as a function of mode number, obtained for the orthorhombic (a) and monoclinic (b) polymorphs of TNT using the TS-vdW and MBD approaches.

As demonstrated above, semi-local DFT approximations, augmented by TS-vdW dispersion interactions, provide good agreement with experiment. Nevertheless, it would be instructive to estimate the performance of more advanced treatments going beyond the pair-wise approximation, especially as these have been shown to affect vibrational spectra in some cases [10,26,47]. We explore this issue by comparing TS-vdW with MBD calculations for both TNT polymorphs. Owing to the use of a different code for this comparison (see section “Computational Approach”), in which tight convergence is more expensive, some difference in the results is encountered already at the TS-vdW level of theory. Nevertheless, Figure 5 clearly establishes that, all other computational details being equal, the difference between TS-vdW and MBD results for the TNT crystal is relatively small, with an average vibrational frequency shift of only 0.15 THz and 0.18 THz for the orthorhombic and monoclinic polymorphs, respectively. Having ruled out MBD interactions as a major issue, one can assume that the remaining theoretical limitations may arise from the underlying exchange–correlation functional itself and/or from anharmonic effects [26,48]. These issues are subject for further research.

**Figure 6:**
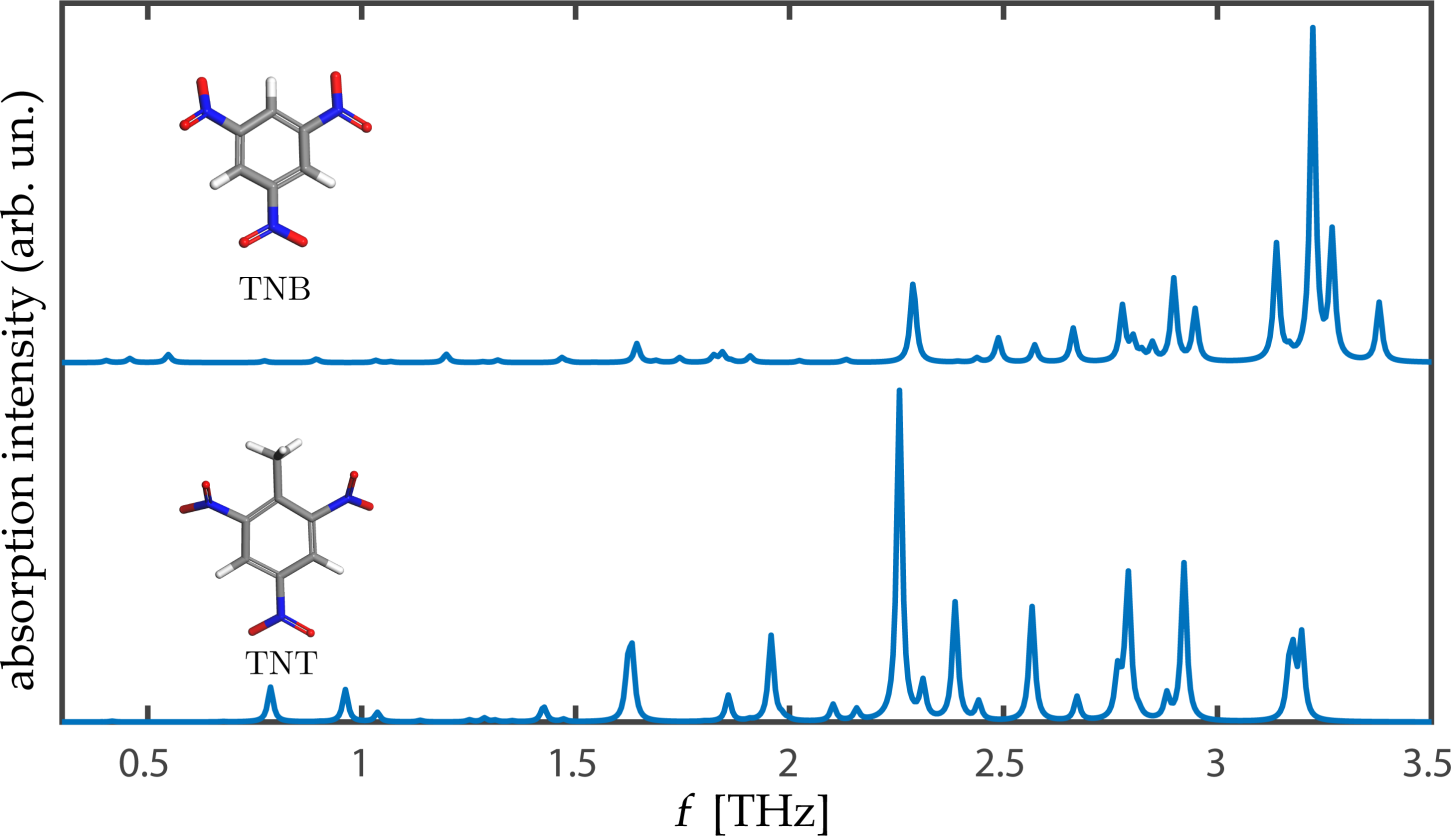
Comparison of computed terahertz spectra, as computed with the PBE+TS-vdW approximation, for the orthorhombic TNB and TNT crystals. Schematic views of the TNT and TNB molecules are given as insets.

Finally, having gained confidence in the predictive power of our approach, we consider 1,3,5-TNB (see structure in Figure 1), for which we are unaware of experimental data in the relevant terahertz range. The TNB molecule differs from TNT merely by the removal of a methyl side group. A computed terahertz spectrum is given in Figure 6, where it is compared to that of orthorombic TNT. The relatively modest chemical modification leaves clear fingerprints in the terahertz spectrum. This demonstrates the significant selectivity of terahertz spectroscopy and the importance of the ability to predict such spectra using advanced computational tools.

## Conclusion

In this article, we have calculated terahertz spectra for the monoclinic and orthorhombic polymorphs of 2,4,6-TNT, using DFT both with and without Tkatchenko–Scheffler pair-wise dispersion interactions. We obtained very good agreement with experimental data upon inclusion of dispersion interactions, whereas lack of dispersion interaction causes sizable shifts in vibrational frequencies and directly affects the spatial character of the vibrational modes. The agreement between theory and experiment allowed us to distinguish between contributions of the two polymorphs to the observed spectrum. Furthermore, we could show that modes in the range from 0 to ca. 3.3 THz bear contributions from both inter- and intramolecular vibrations, with the former dominating below ca. 1.5 THz and the latter primarily involving nitro and methyl groups. Finally, we showed that the theoretical results are little affected by the inclusion many-body dispersion terms for this system, allowing us to present a prediction for the terahertz spectrum of 1,3,5-TNB and showing that a modest chemical modification may result in a markedly different terahertz spectrum.
